# What are we missing? Advantages of more than one viewpoint to estimate fish assemblages using baited video

**DOI:** 10.1098/rsos.171993

**Published:** 2018-05-30

**Authors:** Sasha K. Whitmarsh, Charlie Huveneers, Peter G. Fairweather

**Affiliations:** College of Science and Engineering, Flinders University, Adelaide, South Australia, Australia

**Keywords:** 360° view, baited underwater video, bias, BRUVS, current orientation, monitoring

## Abstract

Counting errors can bias assessments of species abundance and richness, which can affect assessments of stock structure, population structure and monitoring programmes. Many methods for studying ecology use fixed viewpoints (e.g. camera traps, underwater video), but there is little known about how this biases the data obtained. In the marine realm, most studies using baited underwater video, a common method for monitoring fish and nekton, have previously only assessed fishes using a single bait-facing viewpoint. To investigate the biases stemming from using fixed viewpoints, we added cameras to cover 360° views around the units. We found similar species richness for all observed viewpoints but the bait-facing viewpoint recorded the highest fish abundance. Sightings of infrequently seen and shy species increased with the additional cameras and the extra viewpoints allowed the abundance estimates of highly abundant schooling species to be up to 60% higher. We specifically recommend the use of additional cameras for studies focusing on shyer species or those particularly interested in increasing the sensitivity of the method by avoiding saturation in highly abundant species. Studies may also benefit from using additional cameras to focus observation on the downstream viewpoint.

## 1. Introduction

All scientific research methods have inherent biases, and so understanding and mitigating against such biases is essential to the correct interpretation of results [1,2]. In many cases, biases are either ignored or merely acknowledged and, at best, their effects on the findings discussed rather than actively addressed or mitigated against. In particular, many traditional sampling methods such as using fishing gear in the marine realm have limited ways to address potential biases compared to more modern camera-based methods which can provide additional information such as behaviour, habitat and oceanographic conditions (e.g. water clarity and current). Taking action to diminish known biases may enable a better use of scarce resources, including research funds, and improve the accuracy of the data collected [3]. The most likely way in which detection biases are incorporated into ecological data occurs when conducting counts of organisms [2,4]. Such errors can be classified into two types: (i) false negatives (leading to under-estimations of abundance) occur when some individuals cannot be detected and counted or when the whole population of interest is not included within the sampled location and (ii) false positives (leading to over-estimations of abundance) occur through individuals erroneously being counted as being present [1,2,4]. Some studies have previously attempted to address such biases. For example, biases related to species richness estimates within avian communities have been assessed using detection probabilities to determine the number of visits and grid size required to detect rare and common species [5]. In the marine realm, studies have addressed biases associated with fish behaviour seen while scuba-diving during underwater surveys by using bubble-free equipment, i.e. rebreathers [6]. Point-independence analysis has been used to assess the detectability of penguins during ship-based surveys using mark–recapture methods compared to standard strip transects [7]. However, while some methods are suitably studied to address biases, other more novel methods have not had the same level of scrutiny to potential biases inherent in the equipment or experimental design.

With increased use due to availability of low-cost technology, baited remote underwater video stations (BRUVS), comprised of a camera mounted within a frame with bait attached, have become a popular method to assess fish assemblages over the last two decades [8]. They are currently used for a wide variety of purposes [8], ranging from species-specific behavioural information (e.g. [9]) to community analyses, particularly within marine protected areas (e.g. [10]). BRUVS are often chosen due to their non-destructive and non-extractive nature along with their ease of use, archivable footage and replicability [11,12]. BRUVS are suitable to assess a wide range of fish species, predominantly the larger, more mobile species and those often targeted by fishers [11]. However, BRUVS are known to have several biases that are usually only acknowledged rather than being addressed explicitly. For example, BRUVS can be biased towards carnivorous species while being biased against smaller, more cryptic species [11].

Fish behaviour can affect assemblage data obtained using BRUVS if it affects differences in detection probability among species. For example, shy species might exhibit avoidance behaviour (e.g. due to increased predation risk) as a result of the fish activity surrounding the bait [13]. Rare or uncommon species might not be observed on the BRUVS, particularly if they are not piscivorous [13], as non-piscivorous (e.g. herbivorous or planktivorous) species are generally less likely to be attracted to the usually fish-based bait. Species that are territorial or have small home ranges are also less likely to be observed on BRUVS than those that are schooling or highly mobile [14].

Along with detection biases, the standard metric used in BRUVS studies can also bias the relative count obtained. Most BRUVS studies (81% in Whitmarsh *et al*. [8]) use *MaxN* as an abundance measure. *MaxN* is the maximum number of individuals seen within a single frame (for each species) either across the entirety of the sampling period or some time within a video (e.g. each 15 min), and is considered a conservative estimate of relative abundance [15,16]. *MaxN* has been shown under some circumstances to be nonlinearly related to true abundance, such as when abundances at the bait are great but more fish cannot physically fit within the video frame, referred to as screen saturation [17]. Other metrics have been suggested as alternatives to *MaxN,* e.g. *MeanCount* [18], but they also have their own associated biases (e.g. decreased detection probability [19]) and have been shown to be similarly nonlinearly related to true abundance and cause under-estimations for highly abundant species [20].

Currents may also affect fish assemblages observed using BRUVS but there has been little published work investigating their influence on the assemblages observed, despite recommendations for such work to be carried out [21]. Of the research that has been conducted, studies have shown the bait plume will travel downstream and act as an attractant, so that fish will then travel upstream towards the source of the plume [22,23]. Trenkel & Lorance [23] also found that individuals had differing reactions when encountering a bait plume which may further complicate the ability to understand the effect of plume dispersal on fish assemblages. Thus, the direction that the BRUVS faces may influence the number of species observed and so bias abundance estimates through missing individuals or species.

The issues surrounding the potential for counting errors and biases with BRUVS may hinder the uptake of this method by some researchers or cause widespread biases within monitoring datasets. The use of additional cameras, facing in directions other than toward the bait, could increase species richness from observations of shy individuals typically reluctant to approach the bait or through increased abundance estimates due to a greater field of view than with one camera only. This increase in field of view also allows the downstream current direction to be observed, which may lead to sighting more individuals and species as they are likely to swim upstream towards the bait. By increasing the chances of sighting more individuals and species, this modification to BRUVS may help to address some inherent biases and assist in informing the scientific community of changing technological advancements. Thus, the objective of this study is to test whether additional cameras can increase abundance and species richness estimates compared to using a single viewpoint. We aim to (i) study how communities observed on the additional viewpoints differ from the front (bait-facing) viewpoint, and determine whether additional cameras can increase (ii) the observed species richness, (iii) the sightings of shy or infrequently seen species, and (iv) the ability to detect differences in abundance by maximizing *MaxN* estimates, e.g. when screen saturation occurs or by counting individuals not sighted on the bait-facing camera. We also investigate whether our findings are consistent across current directions and different locations.

## 2. Material and methods

This study was conducted within a large temperate gulf, Gulf St Vincent, located in South Australia, Australia. Gulf St Vincent is a relatively shallow (depth less than 30 m) coastal waterbody that acts as a large inverse estuary [24]. A variety of habitats are contained within this gulf including high and low profile rocky reefs, large seagrass meadows, and extensive sandy or finer soft-sediment areas [25]. Interspersed among these are numerous shipwrecks, some of which were purposefully sunk and others the result of historical accidents.

Sampling was conducted at five sites within four habitat types of Gulf St Vincent (electronic supplementary material 1). Aldinga Reef is a high-profile reef system with depths ranging from 4 to 20 m. Long Spit is a shallow (approx. 7 m) sand bank with abundant seagrass meadows (*Posidonia* spp.). The Zanoni (a historically significant shipwreck, sunk in 1865, protected from fishing) and the Barge (vessel purposefully sunk 1.85 km south of the Zanoni to provide an artificial reef where fishing is allowed, alleviating illegal fishing pressure on the Zanoni) are wrecks in deep (18–20 m) soft-sediment habitats, and a site called Near Zanoni (15 m) is situated in soft-sediment habitat 2 km away from the Zanoni, outside the influence of these wrecks and open to fishing. These sites were chosen due to their accessibility and distinct habitat types.

**Figure 1.**
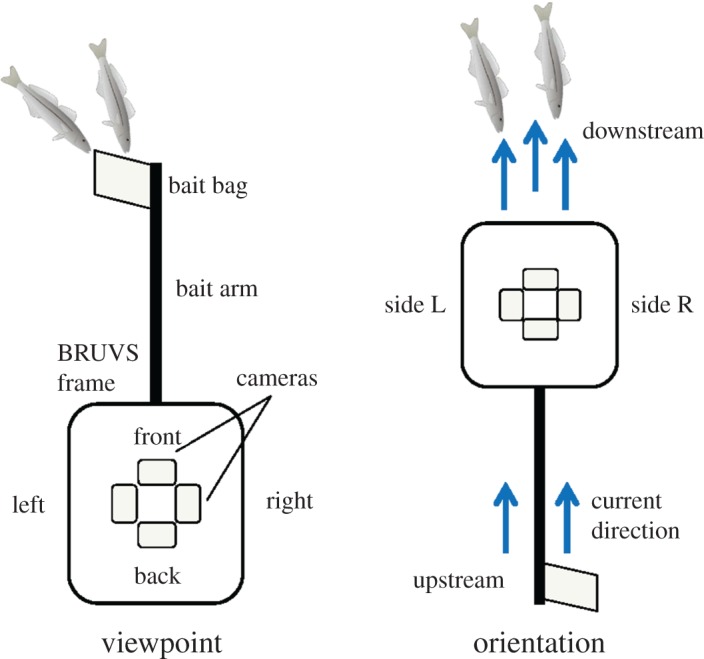
Plan view of the BRUVS set-up used, showing both the Viewpoint and Orientation (in relation to current direction) factors. Each BRUVS unit has one bait bag on a bait arm in front of one camera; the other three cameras do not face any bait. Fish (*Sillaginodes punctatus*) image source: Dieter Tracey, Integration and Application Network, University of Maryland Center for Environmental Science (ian.umces.edu/imagelibrary/).

Custom-built BRUVS units were used, which consisted of a trapezoid metal frame upon which four GoPro Hero 3+ or Hero 4 cameras (set to equivalent settings and tested for field of view differences) were mounted. Each camera faced one of four directions, to the Front (facing the bait bag), each side (Left and Right), and to the Back (figure 1). The opening angle of the camera underwater was calculated to be approximately 94° resulting in practicable 360° views around the BRUVS without overlapping when considering camera spacing. Each BRUVS was baited with 800 g of crushed sardines (*Sardinops sagax*) affixed to the end of the single bait arm within a mesh bag. BRUVS were deployed during daylight hours at each site over a one-month period in the austral summer of 2016. BRUVS were left on the seafloor for 60 min before retrieval. Four replicates were deployed per site. BRUVS were spaced at least 250 m apart. However, some of the BRUVS deployed around the Zanoni and the Barge were slightly closer, to a minimum of 150 m apart (similar to other studies, e.g. [26,27]) because the wrecks were too small to space BRUVS units further apart without being too far from the wreck. Previous studies conducted at the same locations have shown little evidence for identifiable individuals swimming between replicates (S. K. Whitmarsh 2015, unpublished data).

Videos were viewed using the specialized software EventMeasure (www.seagis.com.au). Fish species were identified and then counted using *MaxN*. Species richness, *MaxN* estimates and time of arrival data were calculated for each camera viewpoint. ‘Infrequently seen’ species were classified as species observed fewer than three times across all replicate videos and viewpoints, while ‘shy’ species were classified as those that were observed to be reluctant to approach the bait, did not interact with other species, or seemed reluctant to enter open spaces. Four common species were also chosen to assess how *MaxN* estimates changed when comparing the front and additional cameras: the leatherjacket, *Thamnaconus degeni*; trevally, *Pseudocaranx* spp.; snapper, *Chrysophrys auratus*; and Port Jackson sharks, *Heterodontus portusjacksoni*. These species were selected as they were either abundant schooling species that aggregated in large numbers to feed on the bait (*T. degeni* and *Pseudocaranx* spp.), commonly observed to feed and mill around the BRUVS (*C. auratus*), or in the case of *H. portusjacksoni* have low *MaxN*, but are often present in higher numbers than *MaxN* based on the ability to identify individuals using colour patterns and size.

The video from each viewpoint was processed separately, with *MaxN* estimates recorded for each species. To estimate whether *MaxN* increases when including all four viewpoints, time at *MaxN* on the bait-facing camera was identified, with the *MaxN* observed on the other three viewpoints at that time added to calculate the maximum number of individuals sighted across all four viewpoints while avoiding double-counting. Times between cameras were synched to either the time the BRUVS entered the water or reached the seafloor.

### 2.1. Data analysis

Due to issues with battery longevity, two replicates had viewpoints which did not record for the whole 60 min soak time and thus were excluded from analysis (one from Aldinga Reef and one from Near Zanoni), leaving a final *N* of 18. Most statistical analysis was conducted in PRIMER v. 7 [28] with the PERMANOVA+ add-on [29]. Multi-species assemblage data were transformed using dispersion weighting (by site) [30] and then a square-root transform to account for the variable schooling nature of particular fish species and to down-weight the influence of highly abundant species. Raw and transformed data contributions were visualized using shade plots [31].

To assess whether assemblages differed between viewpoints, a two-factor multivariate PERMANOVA test based on Bray–Curtis similarities was conducted on the factors Site (random factor with five levels) and Viewpoint (fixed factor with four levels). Canonical analysis of principal coordinates (CAP) analysis was also used to test for the influence of Viewpoint alone on assemblages observed and to provide a visual representation via constrained ordination of the similarities within the data. We also used a presence/absence transformation on the multivariate community data to assess whether the species composition alone was affected by Viewpoint and Site using PERMANOVA. Univariate total abundance and species richness data were used to construct Euclidean distance matrices and to subsequently test for differences among viewpoints using the same PERMANOVA model as above. Significant effects were explored further using pair-wise tests on the factor Viewpoint or the Site × Viewpoint interaction.

To assess the influence of current on the assemblages observed, current direction was determined based on the flow of particles and marine plants in front of the cameras. Each viewpoint within a BRUVS deployment was classified in relation to the observed current direction (figure 1). We ran a Pearson Chi-square goodness-of-fit analysis to test whether the distribution of viewpoints across current directions differed from the expected random allocation of 25% (i.e. by chance the Front viewpoint would face the Downstream direction 25% of the time). CAP analyses were then used to assess the effect of current direction on fish assemblage (multivariate) and abundance (univariate).

To assess whether fish activity around the bait served to attract infrequently seen or shy species we used Pearson correlations with Bonferroni probability tests for total fish abundance per replicate against the number infrequently seen or shy species per replicate.

## 3. Results

Overall, this study observed 3601 individuals based on *MaxN* estimates from 46 species, 38 of those being teleost fishes along with four chondrichthyans and four invertebrate species (two decapods, one cephalopod and one echinoderm; electronic supplementary material 2).

### 3.1. Assemblages and abundances of fish

**Figure 2.**
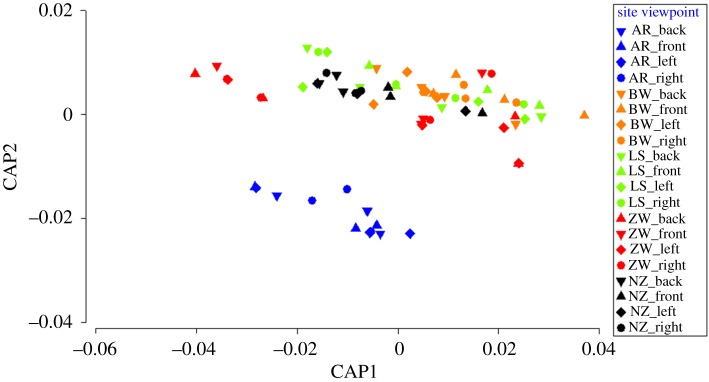
CAP constrained-ordination plot based on multivariate assemblage data showing the separation due to the factor Viewpoint alone (for *m* = 4 axes, allocation success rate = 11.1%). Post-analysis symbols have been modified to also show the influence of sites, where AR, Aldinga Reef; BW, Barge wreck; LS, Long Spit; ZW, Zanoni wreck; NZ, Near Zanoni.

Accounting for both species abundances and composition, there was a significant difference among viewpoints determined from multivariate PERMANOVA (Viewpoint *Pseudo-F* = 2.2571, *p(perm)* = 0.035) but not from CAP analysis (*p* = 0.999, figure 2). The CAP analysis also had a very low allocation-success rate of 11%, implying no distinct differences across viewpoints. Pairwise PERMANOVA tests on the factor Viewpoint were unable to differentiate which pairs had a significant difference (*p* > 0.064, electronic supplementary material 3); however, the smallest *p* values were recorded for the pairs involving the Front viewpoint. For presence/absence-transformed data, no significant differences were detected for taxonomic composition alone (multivariate PERMANOVA *Pseudo-F* = 0.27009, *p(perm)* = 0.974), with these trends also being consistent across the locations studied (i.e. NS Site × Viewpoint interaction *p(perm)* >0.05).

**Figure 3.**
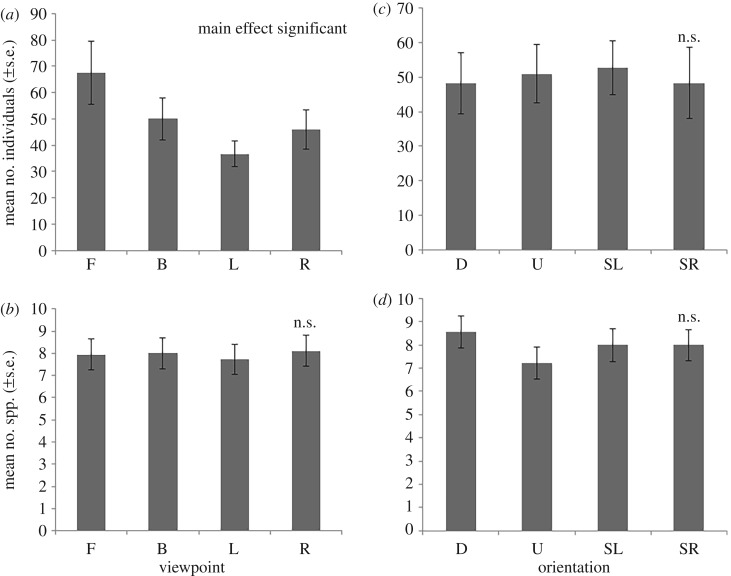
(*a*) Mean (±s.e.) number of total individuals and (*b*) mean (±s.e.) number of species per camera viewpoint for all sites. (*c*) Mean (± s.e.) number of total individuals and (*d*) mean (± s.e.) number of species observed based on the unit's relation to the direction of current for all sites. *N* = 18. F, front; B, back; L, left; R, right; D, downstream; U, upstream; SL, side left; SR, side right.

**Figure 4.**
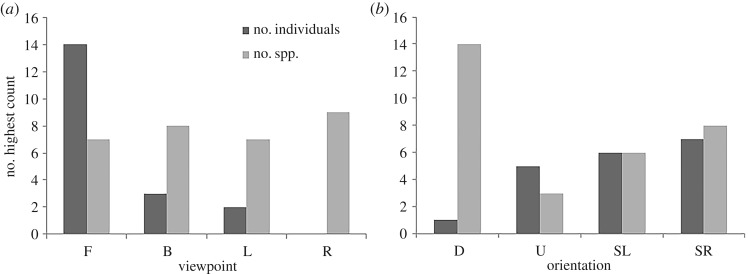
Count of the times (out of 18) that each (*a*) viewpoint or (*b*) current orientation within each replicate had the highest value of either number of species or number of individuals. Draws were counted for all equal Viewpoints, thus the sum of *N* may be greater than 18. F, front; B, back; L, left; R, right; D, downstream; U, upstream; SL, side left; SR, side right.

For total abundance (of pooled species), the Front viewpoint had the highest mean count (figure 3*a*), and univariate PERMANOVA analysis showed similar results as above, with significant differences observed for the factor Viewpoint (*Pseudo-F* = 3.4916, *p(perm)* = 0.045) but pairwise tests were unable to differentiate which pairs were different (*p* > 0.058, electronic supplementary material 3). When considering differences among Viewpoint by Site combinations, some significant differences were able to be identified (table 1), with significant differences being observed between Viewpoints at Near Zanoni and the Barge, where assemblages were dominated by the highly abundant schooling species *T. degeni* (electronic supplementary material 4). The Front view was also the most likely to have the highest abundance of any viewpoint within a replicate deployment (figure 4*a*).

**Table 1.** Pairwise PERMANOVA tests of the Viewpoint factor listed by site for univariate PERMANOVA analysis of total individuals per viewpoint, where italic values are significant (*α* = 0.05) Monte Carlo *p* values whereas n.s. are non-significant (*p* > 0.05). Comparisons between pairs not involving the Front viewpoint (i.e. Back versus Left, Left versus Right and Back versus Right) are not shown here because they were non-significant (all *p* > 0.12).

	site				
pairwise comparison of viewpoints	Aldinga	Barge	Long Spit	Zanoni	Near Zanoni
Front versus Back	n.s.	n.s.	n.s.	n.s.	*0.035*
Front versus Left	n.s.	*0.008*	n.s.	n.s.	*0.003*
Front versus Right	n.s.	n.s.	n.s.	n.s.	*0.008*

Because we were unable to direct each BRUVS to face a particular current direction, we had an uneven distribution of viewpoint to current orientations, with the Front view facing the Downstream direction on three occasions, the Back on four, the Left on six, and the Right on five occasions (total *N* = 18). However, this distribution was not significantly different from the expected random placement of 25% in each direction (Pearson Chi-square = 1.111, *p* = 0.774, d.f. = 3). CAP detected no significant difference for the species abundance and composition observed from the different viewpoints in relation to the Current Direction (*p* = 0.981, allocation success rate = 12.5%). Similarly, there was also no significant difference for total abundance of individuals in relation to current (CAP *p* = 0.602, allocation success rate = 4.2%; figure 3*c*).

### 3.2. Species richness

There were no significant differences among viewpoints in the number of species seen per Viewpoint (figure 3*b*; univariate PERMANOVA *Pseudo-F* = 0.27368, *p(perm)* = 0.84) but cumulatively there was a moderate increase in the observed number of species when more cameras were used (mean ± s.e. = 7.9 ± 3.0 for one camera compared to 9.8 ± 3.5 for four cameras). When comparing the viewpoints within each replicate, there was also no clear trend for any particular viewpoint to show the highest number of species (figure 4*a*).

There was no significant difference in the number of species observed in relation to the Current Direction factor (*p* > 0.05, allocation success rate = 27.8%; figure 3*d*). It was, however, more common for the Downstream direction to observe the most species compared to the other orientations within each replicate (figure 4*b*). There was also a trend for the Downstream direction to observe a species for the first time within a replicate deployment with 48% of first sightings occurring in that orientation compared to 9% for the Upstream and 21% for each side. The Downstream orientation also had the most occurrences of a species first appearing compared to other orientations with 12 out of 18 replicates (67%) observing the most first-sightings Downstream of any orientation, while Upstream never had the most and the Side Right and Side Left had the most on three and four replicates, respectively.

### 3.3. Infrequently seen or shy species

**Figure 5.**
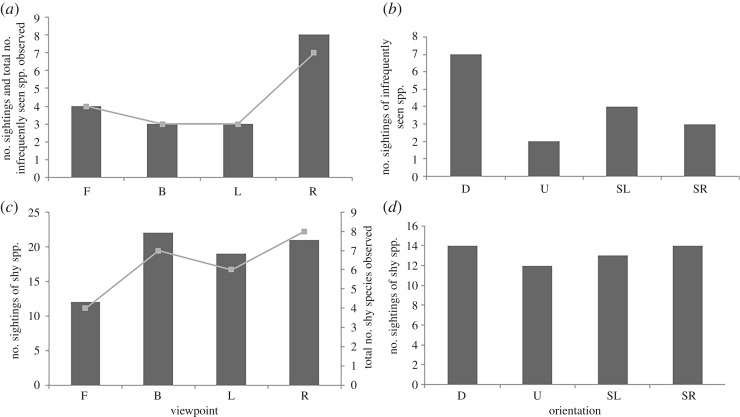
The total number of sightings of infrequently seen species for (*a*) Viewpoint (shown as bars), with the total number of infrequently seen species observed from each viewpoint (shown as a line) and (*b*) current Orientation. The total number of sightings of shy species for (*c*) Viewpoint (shown as bars), with the total number of shy species observed from each viewpoint (shown as a line) and (*d*) current Orientation. F, front; B, back; L, left; R, right; D, downstream; U, upstream; SL, side left; SR, side right.

Ten species out of the total 46 were classified as infrequently seen (table 2) and similar numbers of sightings of infrequently seen species were observed across viewpoints, with slightly more being observed on the right side than other viewpoints (figure 5*a*). There was, however, a trend for more of these species to be observed in the Downstream direction (figure 5*b*), with approximately 4.5% of total species sightings that were infrequently seen for the Downstream compared to 1.5% for the Upstream, 2.1% and 2.8% for the Side Right and Side Left, respectively.

**Table 2.** A list of the infrequently seen and/or shy species and the number of times each was observed from the 72 videos.

species	infrequently seen	shy	no. of times observed
*Acanthaluteres brownii*	X		2
*Chelmonops curiosus*	X		2
*Leptomithrax gaimardii*	X		1
*Olisthops cyanomelas*	X		1
*Omegophora armilla*	X	X	2
*Parma victoriae*	X		2
*Platycephalus speculator*	X		3
*Sepioteuthis australis*	X		3
*Siphonognathus radiates*	X	X	1
*Coscinasterias muricata*	X		1
*Aracana ornata*		X	5
*Haletta semifasciata*		X	9
*Neoodax balteatus*		X	8
*Parapercis haackei*		X	35
*Siphamia cephalotes*		X	10
*Siphonognathus* sp.		X	4

Eight species were considered to be shy (table 2). There was a consistent trend for both lower numbers of sightings of shy species on the front camera and fewer overall shy species observed from the front viewpoint compared to all other viewpoints (figure 5*c*). There appeared to be no influence of current direction on the number of sightings of shy species (figure 5*d*). Pearson correlations and Bonferroni probabilities showed that neither shy species (*r* = 0.005 and *p* = 0.983) nor infrequently seen species (*r* = −0.463 and *p *= 0.053) were significantly correlated with total fish abundance around the BRUVS.

### 3.4. *MaxN* estimates

**Figure 6.**
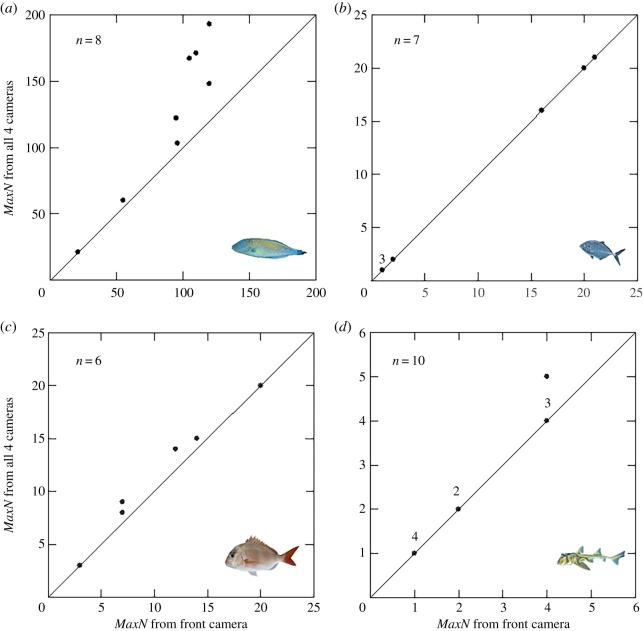
*MaxN* estimates for four species for the front camera only compared to all cameras combined for the species: (*a*) leatherjacket *Thamnaconus degeni*; (*b*) trevally *Pseudocaranx* spp.; (*c*) snapper *Chrysophrys auratus*; (*d*) Port Jackson shark *Heterodontus portusjacksoni*. Only replicates that had a sighting of the target species on the front camera were included in the analysis. Each point indicates a single replicate deployment with numbers above the points indicating the number of any overlapping points. Photo source: Rudie H. Kuiter, except for *C. auratus* (Copyright © State of New South Wales).

Similar *MaxN* estimates were gained from front-only estimates compared to the cumulative total for all cameras for both *Pseudocaranx* spp. and *H. portusjacksoni* (figure 6*b*,*d*). For 4 out of 6 replicates that observed *C. auratus*, the combined estimate was slightly higher than the front-only estimate (figure 6*c*). *Thamnaconus degeni* showed the greatest difference between front and combined estimates for *MaxN* (figure 6*a*), with some replicates having up to 60% more individuals counted using the combined estimates than the front-only estimate but this only occurred at the highest densities.

## 4. Discussion

The use of three additional viewpoints with BRUVS allowed us to better understand and mitigate potential biases associated with the single-camera method. The additional viewpoints were able to reduce the likelihood of false negatives by increasing the chance of observing shy and infrequently seen species, thus sampling a larger proportion of the total population in the vicinity of the BRUVS unit. The additional viewpoints also provided an increase in sensitivity for abundance estimates when assemblages became saturated on the front camera.

There were detectable differences among the front and additional viewpoints with most of these differences being driven by the higher abundance of some species on the front viewpoint. Attracted individuals are likely to be enticed to the bait bag and congregate within this vicinity to feed, increasing the abundance seen from the front viewpoint compared to those without bait [21,32]. This pattern seems particularly prevalent for schooling species, especially those that commonly feed upon the bait, such as *T. degeni* (figure 6*a*).

The additional viewpoints had little impact on overall observed species richness, with a trend for additional cameras to only slightly increase the number of species observed. While the increase in species numbers is small, there are situations where it could be necessary for scientists to observe the maximum diversity within an area (e.g. to assess marine protected areas). Monitoring programmes focused on protected areas desire the maximal biodiversity of these areas to be recorded [12], thus enabling researchers to know which species may be benefiting from protection and conservation efforts, and having an ability to track assemblages over time.

Currents may also play an important role in determining the numbers of species observed, with the downstream direction the most likely to observe the highest species richness within a replicate deployment. The attraction of individuals towards the bait along the bait plume is likely to be the reason that more species were observed in the downstream direction. Fish (and other animals) attracted to BRUVS by the olfactory stimulus of the bait plume travel upstream towards the source [21] and are, therefore, more likely to be observed in the downstream direction. Corroborating this, time-of-first-arrival data implied that most fish are following the bait plume to the BRUVS because the downstream direction is usually the first direction to detect a species and thus has more first sightings of species compared to other orientations and viewpoints. Therefore, facing the BRUVS downstream using divers, a modified frame design, or 360° BRUVS may also lead to a reduction in false negatives. Similar to current, proximity to structures (e.g. reef) may also affect the assemblages observed [22,23], and by using additional viewpoints researchers can ensure any viewpoints facing structures can also be adequately sampled.

While there was no preference for any viewpoint shown by infrequently seen species, these species were more often observed in the downstream current direction. Infrequently seen species may be less common within an area or behaviourally disinclined to approach the BRUVS unit and thus less likely to be observed by the camera. Regardless of the reason for species being seen infrequently, there may be times when a comprehensive species list is particularly important, such as when researchers are interested in documenting the biodiversity of an area. Such is the case when assessing marine protected area performance or when targeting selected species such as in the Global FinPrint project (https://globalfinprint.org/), a large-scale study with the aim of assessing populations of sharks and rays in coral reef areas. By using sampling approaches such as additional viewpoints or deliberately facing a BRUVS downstream, researchers would be better able to use BRUVS to sample certain assemblages (such as those species that are seen infrequently) and thus reduce bias.

In contrast, shy species had a tendency to prefer viewpoints that were not the front. The high levels of activity surrounding the bait might have affected the numbers of shy species observed by the front viewpoint. Species identified as shy are often smaller and may avoid the activity around bait or are disinclined to approach due to the bait type not comprising their typical diet. Researchers interested in shyer species may benefit from extra cameras at additional viewpoints to increase the likelihood of observing these shy species and also similarly reduce the prevalence of false negatives.

A major benefit of the additional viewpoints is the ability to maximize the *MaxN* estimates for abundance and increase the sensitivity of *MaxN*, allowing for detecting differences due to factors that are not detectable using a single viewpoint. *MaxN* has been reported to have a nonlinear relationship with true abundance, particularly for highly abundant species that can reach a saturation point, where more individuals cannot fit within the field of view [17,18]. We have found similar results from our study as by having the extra viewpoints we were able to count more individuals within the areas surrounding the BRUVS. This enabled a better representation of the abundance of a given species, especially in cases where a species was highly abundant and thus more likely to have an abundance nonlinearly related to *MaxN* estimates. Therefore, additional viewpoints were able to reduce biases such as false negatives. This may be useful in studies requiring more sensitive abundance estimates such as in marine protected areas, where researchers may wish to detect any abundance differences between closed and open areas, or to conduct stock assessments. Maximizing *MaxN* estimates was most effective for highly abundant species, such as *T. degeni*. For species with lower abundances or without a tendency to mill around the BRUVS, there are fewer benefits from using additional viewpoints to maximize *MaxN*. For example, often there were no individuals occurring on any of the other viewpoints when *MaxN* occurred at the front for *Pseudocaranx* spp. or *H. portusjacksoni* (figure 6). These results are similar to others [19], and further emphasize that those species which have lower abundances are less affected by the nonlinearity issues with *MaxN*.

A different method of maximizing relative abundance estimates could also be inferred from using multiple viewpoints. The additional viewpoints can allow researchers to identify times when the highest number of individuals can be observed across all viewpoints, rather than when *MaxN* is reached on the front viewpoint. It would, however, be very time consuming with the present technology to identify when such a hypothetical maximum occurred. It could nevertheless be calculated by either watching the four viewpoint videos simultaneously to find the highest *MaxN* across all viewpoints (similar to what is done by those using stitched-together 360° videos, e.g. [20]) or to record fish numbers continuously as they enter and leave the field of view and subsequently analyse the data to find the highest *MaxN*. If advances were made in the automation of software for *MaxN* analysis, better estimates could be calculated using additional viewpoints. Advancements also in the use of commercially available 360° video units have made it possible to use stitched-together 360° viewpoints to calculate abundances [20]. One of the benefits of using the method of the present study to maximize *MaxN* is that it is relatively quick and only requires approximately 15 min to calculate for the first species within a replicate with subsequent species taking a shorter amount of time due to less file opening and calculating synchronization times for the videos. Comparatively, to fully analyse the videos using standard analysis techniques with non-automated software, each additional camera increases video processing time. The additional cameras also add some extra capital cost to the project for equipment and may be logistically more challenging to organize in the field, but newly commercially available 360° units are becoming increasingly more affordable.

Findings from this study are applicable to other methods using fixed viewpoints such as terrestrially based camera traps. For example, O'Connor *et al*. [33] investigated the use of additional cameras to survey wildlife and mesopredator activity in North America and found similar results to our study on marine fishes. Common species were equally detected using one or several cameras but infrequently seen species were more likely to be observed through the addition of extra cameras [33]. Recent studies have also promoted the use of remote cameras to form a global network for monitoring biodiversity [34]. It is likely that as technology improves and costs continue to fall for photography and videography equipment, then more studies and monitoring programmes will use such methods.

In conclusion, the additional cameras enabled us to better understand the biases associated with counting abundance and richness from a single viewpoint. We were able to reduce the effect of one type of counting error, false negatives, by observing additional species and individuals not present on the front viewpoint. In particular, the extra viewpoints are useful for observing infrequently seen and shy species through the ability to view the downstream current direction and viewpoints not containing the bait and associated activity. While all optical-based surveys are likely to benefit, studies incorporating additional viewpoints may particularly benefit from use of automated video analyses that dramatically cut down video processing time (e.g. through machine learning). Additionally, 360° views are useful for those studies which are likely to have a high saturation of individuals on the front camera, and studies requiring species list and diversity estimates as comprehensive as possible.

## Supplementary Material

Electronic supplementary material 1: Map of study sites

## Supplementary Material

Electronic supplementary material 2: Species list

## Supplementary Material

Electronic supplementary material 3: Pairwise PERMANOVA results

## Supplementary Material

Electronic supplementary material 4: SIMPER results

## Data accessibility

Primary data are accessible through the following DOI on the Flinders University Data Research Repository: https://dx.doi.org/10.4226/86/5a1f7e71b6726.

## References

[1] Tyre AJ, Tenhumberg B, Field SA, Niejalke D, Parris K, Possingham HP 2003 Improving precision and reducing bias in biological surveys: estimating false-negative error rates. Ecol. Appl. 13, 1790–1801. (doi:10.1890/02-5078)

[2] Elphick CS 2008 How you count counts: the importance of methods research in applied ecology. J. Appl. Ecol. 45, 1313–1320. (doi:10.1111/j.1365-2664.2008.01545.x)

[3] Moore AL, McCarthy MA, Parris KM, Moore JL 2014 The optimal number of surveys when detectability varies. PLoS ONE 9, e115345 (doi:10.1371/journal.pone.0115345) 25526514 10.1371/journal.pone.0115345 PMC4272285

[4] Dénes FV, Silveira LF, Beissinger SR 2015 Estimating abundance of unmarked animal populations: accounting for imperfect detection and other sources of zero inflation. Methods Ecol. Evol. 6, 543–556. (doi:10.1111/2041-210X.12333)

[5] Sliwinski M, Powell L, Koper N, Giovanni M, Schacht W 2015 Research design considerations to ensure detection of all species in an avian community. Methods Ecol. Evol. 7, 456–462. (doi:10.1111/2041-210X.12506)

[6] Lindfield SJ, Harvey ES, McIlwain JL, Halford AR 2014 Silent fish surveys: bubble-free diving highlights inaccuracies associated with SCUBA-based surveys in heavily fished areas. Methods Ecol. Evol. 5, 1061–1069. (doi:10.1111/2041-210X.12262)

[7] Southwell C, Low M 2009 Black and white or shades of grey? Detectability of Adélie penguins during shipboard surveys in the Antarctic pack-ice. J. Appl. Ecol. 46, 136–143. (doi:10.1111/j.1365-2664.2008.01584.x)

[8] Whitmarsh SK, Fairweather PG, Huveneers C 2017 What is Big BRUVver up to? Methods and uses of baited underwater video. Rev. Fish Biol. Fish. 27, 53–73. (doi:10.1007/s11160-016-9450-1)

[9] Santana-Garcon J, Leis J, Newman S, Harvey E 2014 Presettlement schooling behaviour of a priacanthid, the Purplespotted Bigeye *Priacanthus tayenus* (*Priacanthidae: Teleostei*). Environ. Biol. Fishes 97, 277–283. (doi:10.1007/s10641-013-0150-6)

[10] Whitmarsh S, Fairweather P, Brock D, Miller D 2014 Nektonic assemblages determined from baited underwater video in protected versus unprotected shallow seagrass meadows on Kangaroo Island, South Australia. Mar. Ecol. Prog. Ser. 503, 205–218. (doi:10.3354/meps10733)

[11] Colton M, Swearer S 2010 A comparison of two survey methods: differences between underwater visual census and baited remote underwater video. Mar. Ecol. Prog. Ser. 400, 19–36. (doi:10.3354/meps08377)

[12] Murphy HM, Jenkins GP 2010 Observational methods used in marine spatial monitoring of fishes and associated habitats: a review. Mar. Freshw. Res. 61, 236–252. (doi:10.1071/MF09068)

[13] Lowry M, Folpp H, Gregson M, Suthers I 2012 Comparison of baited remote underwater video (BRUV) and underwater visual census (UVC) for assessment of artificial reefs in estuaries. J. Exp. Mar. Biol. Ecol. 416, 243–253. (doi:10.1016/j.jembe.2012.01.013)

[14] Watson D, Harvey E, Fitzpatrick B, Langlois T, Shedrawi G 2010 Assessing reef fish assemblage structure: how do different stereo-video techniques compare? Mar. Biol. 157, 1237–1250. (doi:10.1007/s00227-010-1404-x)

[15] Cappo M, Harvey E, Shortis M 2007 Counting and measuring fish with baited video techniques—an overview *Proc. 2006 Australian Society of Fish Biology Conference and Workshop: Cutting edge Technologies in Fish and Fisheries Science, Hobart, Australia, August 2006* (eds Lyle JM, Furlani DM, Buxton CD), pp. 101–114. Hobart, Australia: ASFB.

[16] Farnsworth KD, Thygesen UH, Ditlevsen S, King NJ 2007 How to estimate scavenger fish abundance using baited camera data. Mar. Ecol. Prog. Ser. 350, 223–234. (doi:10.3354/meps07190)

[17] Stobart B, Díaz D, Álvarez F, Alonso C, Mallol S, Goñi R 2015 Performance of baited underwater video: does it underestimate abundance at high population densities? PLoS ONE 10, e0127559 (doi:10.1371/journal.pone.0127559) 26010738 10.1371/journal.pone.0127559 PMC4444247

[18] Schobernd ZH, Bacheler NM, Conn PB 2014 Examining the utility of alternative video monitoring metrics for indexing reef fish abundance. Can. J. Fish. Aquat. Sci. 71, 464–471. (doi:10.1139/cjfas-2013-0086)

[19] Campbell MD, Pollack AG, Gledhill CT, Switzer TS, DeVries DA 2015 Comparison of relative abundance indices calculated from two methods of generating video count data. Fish. Res. 170, 125–133. (doi:10.1016/j.fishres.2015.05.011)

[20] Kilfoil JP, Wirsing AJ, Campbell MD, Kiszka JJ, Gastrich KR, Heithaus MR, Zhang Y, Bond ME 2017 Baited remote underwater video surveys undercount sharks at high densities: insights from full-spherical camera technologies. Mar. Ecol. Prog. Ser. 585, 113–121. (doi:10.3354/meps12395)

[21] Taylor MD, Baker J, Suthers IM 2013 Tidal currents, sampling effort and baited remote underwater video (BRUV) surveys: are we drawing the right conclusions? Fish. Res. 140, 96–104. (doi:10.1016/j.fishres.2012.12.013)

[22] Bacheler NM, Berrane DJ, Mitchell WA, Schobernd CM, Schobernd ZH, Teer BZ, Ballenger JC 2014 Environmental conditions and habitat characteristics influence trap and video detection probabilities for reef fish species*.* Mar. Ecol. Prog. Ser. 517, 1–14. (doi:10.3354/meps11094)

[23] Trenkel VM, Lorance P 2011 Estimating *Synaphobranchus kaupii* densities: contribution of fish behaviour to differences between bait experiments and visual strip transects. Deep Sea Res. I Oceanogr. Res. Pap. 58, 63–71. (doi:10.1016/j.dsr.2010.11.006)

[24] Bye J, Kampf J 2008 Physical oceanography. In Natural history of Gulf St Vincent (eds Shepherd SA, Bryars S, Kirkegaard IR, Harbison P, Jennings JT), pp. 56–71. Adelaide, Australia: Royal Society of South Australia Inc.

[25] Tanner J 2005 Three decades of habitat change in Gulf St. Vincent, South Australia. Trans. R. Soc. S. Aust. 129, 65–73.

[26] Gladstone W, Lindfield S, Coleman M, Kelaher B 2012 Optimisation of baited remote underwater video sampling designs for estuarine fish assemblages. J. Exp. Mar. Biol. Ecol. 429, 28–35. (doi:10.1016/j.jembe.2012.06.013)

[27] Lindfield SJ, McIlwain JL, Harvey ES 2014 Depth refuge and the impacts of SCUBA spearfishing on coral reef fishes. PLoS ONE 9, e92628 (doi:10.1371/journal.pone.0092628) 24663400 10.1371/journal.pone.0092628 PMC3963921

[28] Clarke K, Gorley R 2015 PRIMER v7: user manual/tutorial. Plymouth, UK: PRIMER-E Ltd.

[29] Anderson M, Gorley R, Clarke K 2008 PERMANOVA+ for PRIMER: guide to software and statistical methods. Plymouth, UK: PRIMER-E Ltd.

[30] Clarke K, Chapman M, Somerfield P, Needham H 2006 Dispersion-based weighting of species counts in assemblage analyses. Mar. Ecol. Prog. Ser. 320, 11–27. (doi:10.3354/meps320011)

[31] Clarke KR, Tweedley JR, Valesini FJ 2014 Simple shade plots aid better long-term choices of data pre-treatment in multivariate assemblage studies. J. Mar. Biol. Assoc. UK 94, 1–16. (doi:10.1017/S0025315413001227)

[32] Harasti D, Malcolm H, Gallen C, Coleman MA, Jordan A, Knott NA 2015 Appropriate set times to represent patterns of rocky reef fishes using baited video. J. Exp. Mar. Biol. Ecol. 463, 173–180. (doi:10.1016/j.jembe.2014.12.003)

[33] O'Connor KM, Nathan LR, Liberati MR, Tingley MW, Vokoun JC, Rittenhouse TAG 2017 Camera trap arrays improve detection probability of wildlife: investigating study design considerations using an empirical dataset. PLoS ONE 12, e0175684 (doi:10.1371/journal.pone.0175684) 28422973 10.1371/journal.pone.0175684 PMC5396891

[34] Steenweg R et al. 2017 Scaling-up camera traps: monitoring the planet's biodiversity with networks of remote sensors. Front. Ecol. Environ. 15, 26–34. (doi:10.1002/fee.1448)

