# Supplementary material for: What are we missing? Advantages of more than one viewpoint to estimate fish assemblages using baited video
Source: R Soc Open Sci. 2018 May 30;5(5):171993. doi: 10.1098/rsos.171993 (PMC5990793; doi:10.1098/rsos.171993)
Supplement: Electronic supplementary material 1: Map of study sites [file rsos171993supp1.pdf]

Electronic supplementary material 1

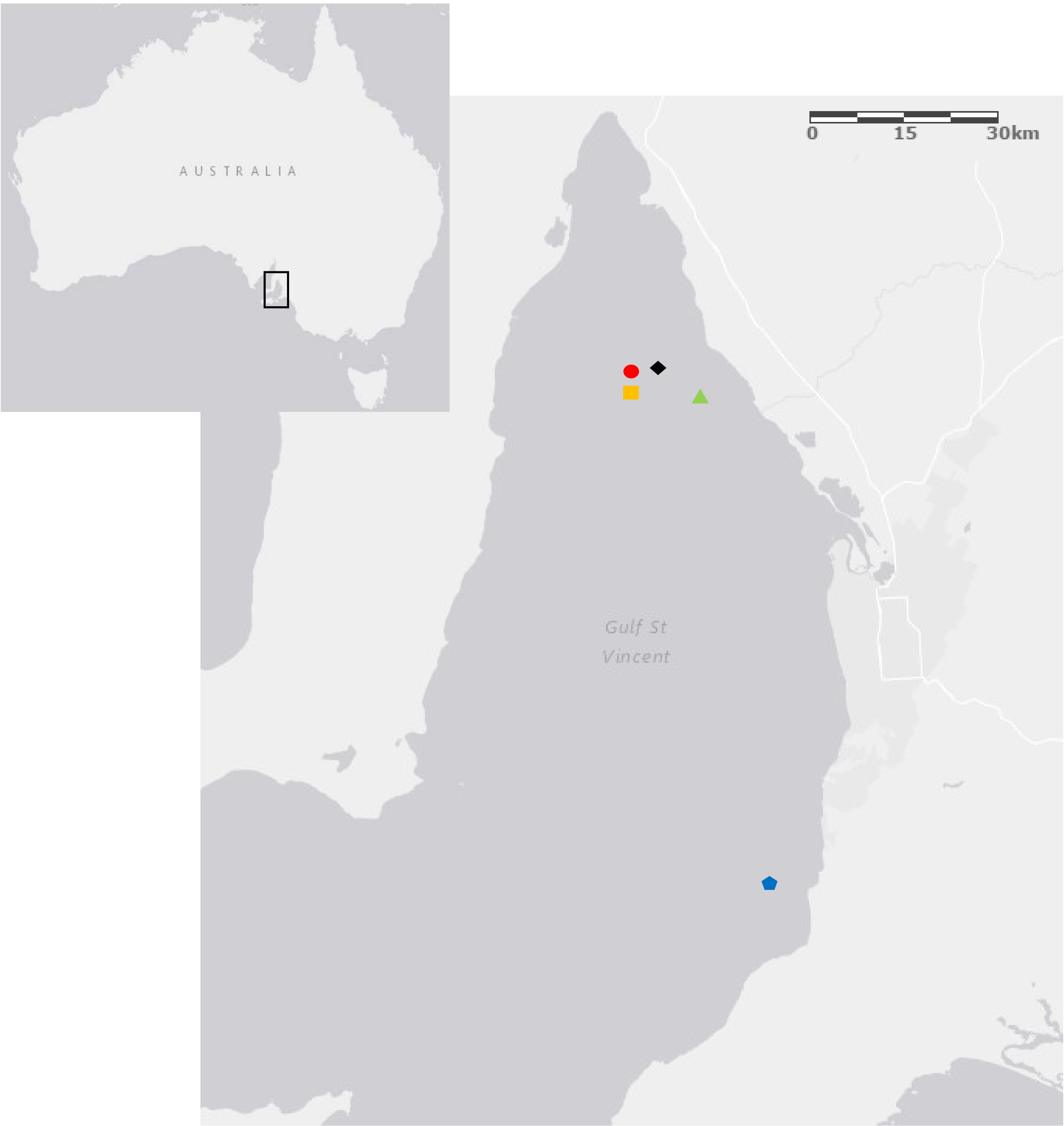

Figure S1: Map showing the sites where BRUVS were deployed. The blue pentagon indicates Aldinga Reef (S35.27360 E138.43265), the green triangle Long Spit (S34.56461 E138.22672), the orange square the Barge wreck (S34.52841 E138.06356), the red circle the Zanoni wreck (S34.51163 E138.06368) and the black diamond Near Zanoni (S34.51496 E138.08525).
