# Supplementary material for: What are we missing? Advantages of more than one viewpoint to estimate fish assemblages using baited video
Source: R Soc Open Sci. 2018 May 30;5(5):171993. doi: 10.1098/rsos.171993 (PMC5990793; doi:10.1098/rsos.171993)
Supplement: Electronic supplementary material 2: Species list [file rsos171993supp2.pdf]

Electronic supplementary material 2

Table S2: Presence of taxa at each site, with an X indicating at least one sighting, for species observed using 360° view BRUVS. *Pseudocaranx* spp. consisted of two similar co-occurring species, *Pseudocaranx wrighti* and *Pseudocaranx georgianus*.

Taxa and Taxonomic authority	Aldinga Reef	Barge	Long Spit	Near Zanoni	Zanoni
**Teleosts**					
Acanthaluteres brownii (Richardson 1846)	X				
Acanthaluteres vittiger (Castelnau 1873)	X		X		
Aracana ornata (Gray 1838)				X	
Arripis georgianus (Valenciennes 1831)	X		X		
Austrolabrus maculatus (Macleay 1881)	X				
Cheilodactylus nigripes Richardson 1850	X				
Chelmonops curiosus Kuiter 1986					X
Dactylophora nigricans (Richardson 1850)	X				X
Haletta semifasciata (Valenciennes 1840)			X		
Kyphosus sydneyanus (Günther 1886)	X				
Meuschenia freycineti (Quoy & Gaimard 1824)			X		X
Meuschenia hippocrepis (Quoy & Gaimard 1824)	X				
Neoodax balteatus (Valenciennes 1840)			X		
Notolabrus parilus (Richardson 1850)	X				
Notolabrus tetricus (Richardson 1840)	X				
Olisthops cyanomelas (Richardson 1850)	X				
Omegophora armilla (McCulloch & Waite 1915)	X				
Chrysophrys auratus (Forster 1801)	X	X			X
Parapercis haackei (Steindachner 1884)	X	X		X	X
Parequula melbournensis (Castelnau 1872)	X	X			
Parma victoriae (Günther 1863)	X				
Pelates octolineatus (Jenyns 1840)		X	X	X	X
Pentaceropsis recurvirostris (Richardson 1845)	X				
Pictilabrus laticlavius (Richardson 1839)	X				
Platycephalus speculator Klunzinger 1872		X			
Pseudocaranx spp.	X	X			X
Scobinichthys granulatus (White 1790)	X		X	X	
Scorpis aequipinnis Richardson 1848	X				
Sillaginodes punctatus (Cuvier 1829)	X		X	X	
Siphamia cephalotes (Castelnau 1875)			X		
Siphonognathus sp.	X				
Siphonognathus radiatus (Quoy & Gaimard 1834)			X		
Sphyraena novaehollandiae Günther 1860			X		
Thamnaconus degeni (Regan 1903)		X		X	X
Tilodon sexfasciatus (Richardson 1842)	X				
Torquigener pleurogramma (Regan 1903)			X		
Trachurus novaezelandiae Richardson 1843			X	X	X
Upeneichthys vlamingii (Cuvier 1829)	X	X	X	X	

**Chondrichthyans**					
Bathytoshia brevicaudata (Hutton 1875)			X		
Heterodontus portusjacksoni (Meyer 1793)		X	X	X	X
Notorynchus cepedianus (Péron 1807)		X			
Trygonorrhina dumerilii (Castelnau 1873)		X	X	X	X
**Invertebrates**					
Coscinasterias muricata Verrill, 1867	X				
Leptomithrax gaimardii (H. Milne Edwards, 1834)				X	
Portunus armatus (A. Milne-Edwards, 1861)		X	X	X	X
Sepioteuthis australis Quoy & Gaimard, 1832			X		
**Total**	**25**	**11**	**17**	**10**	**11**
