# Supplementary material for: What are we missing? Advantages of more than one viewpoint to estimate fish assemblages using baited video
Source: R Soc Open Sci. 2018 May 30;5(5):171993. doi: 10.1098/rsos.171993 (PMC5990793; doi:10.1098/rsos.171993)
Supplement: Electronic supplementary material 3: Pairwise PERMANOVA results [file rsos171993supp3.pdf]

Electronic supplementary material 3

Table S3: Pairwise PERMANOVA tests of the Viewpoint factor for multivariate analysis of assemblages and univariate analysis of total individuals per viewpoint. Unique permutations ranged from 982-996.

	**Multivariate**		**Univariate**	
**Groups**	***t***	**p(perm)**	***t***	***p*(perm)**
Back, Front	1.646	0.092	1.238	0.277
Back, Left	0.031	0.846	3.260	0.058
Back, Right	0.973	0.524	0.882	0.444
Front, Left	1.836	0.076	2.140	0.104
Front, Right	2.042	0.064	2.021	0.104
Left, Right	1.171	0.326	2.271	0.091
