# Supplementary material for: What are we missing? Advantages of more than one viewpoint to estimate fish assemblages using baited video
Source: R Soc Open Sci. 2018 May 30;5(5):171993. doi: 10.1098/rsos.171993 (PMC5990793; doi:10.1098/rsos.171993)
Supplement: Electronic supplementary material 4: SIMPER results [file rsos171993supp4.pdf]

Electronic supplementary material 4

Table S4: SIMPER results showing the average similarity of assemblages from each site and the dominant taxa (in descending order) with their contribution to that overall site similarity.

**Site**	**Average similarity (%)**	**Dominant taxa**	**Average abundance**	**% contribution to similarity**
Aldinga	38	*Upeneichthys vlamingii*	2.75	15.2
		Pseudocaranx spp.	4.75	12.3
		Austrolabrus maculatus	1.92	11.9
		Notolabrus parilus	1.08	8.0
		Chrysophrys auratus	2.42	7.9
		Parequula melbournensis	1.17	7.9
		Tilodon sexfasciatus	0.83	6.9
Barge	53	*Thamnaconus degeni*	49.31	70.9
Long Spit	39	*Siphamia cephalotes*	17.75	27.7
		Heterodontus portusjacksoni	2.19	14.7
		Pelates octolineatus	2.13	11.6
		Sillaginodes punctatus	1.25	8.2
		Portunus armatus	1.63	7.8
Near Zanoni	69	*Thamnaconus degeni*	59.00	60.6
		Pelates octolineatus	27.58	28.2
Zanoni	33	*Chrysophrys auratus*	6.19	46.4
		Pseudocaranx spp.	6.63	20.0
		Trachurus novaezelandiae	2.19	11.2
